# Prevalence, risky behaviors, and antimicrobial resistance of urinary tract infections in pregnant women: A study in Jordan

**DOI:** 10.1097/MD.0000000000041986

**Published:** 2025-04-25

**Authors:** Asma Bakleezi, Esra’ O. Taybeh, Abdalrahman Binodeh, Alaa A. Alsharif, Meshari Alhamed, Abdallah Y. Naser

**Affiliations:** a Department of Applied Pharmaceutical Sciences and Clinical Pharmacy, Faculty of Pharmacy, Isra University, Amman, Jordan; b Dr. Jamil Al Totanji Hospital, Jordanian Ministry of Health, Amman, Jordan; c Department of Pharmacy Practice, College of Pharmacy, Princess Nourah bint Abdulrahman University, Riyadh, Saudi Arabia; d Department of Emergency Medicine, Ministry of National Guard Health Affairs, Riyadh, Saudi Arabia; e King Abdullah International Medical Research Center, Riyadh, Saudi Arabia.

**Keywords:** adherence, practices, pregnancy, urinary trac infection

## Abstract

Urinary tract infection (UTI) is prevalent among pregnant women, emerging as the most frequent type of infection during pregnancy. This study aimed to reveal UTI prevalence in Jordan, identify risk practices, assess antibiotic adherence, and evaluate UTI recurrence among pregnant women. A prospective observational cohort study, conducted from January to July 2023, was employed to Urinary tract infections (UTIs) during pregnancy. Participants were recruited during routine visits to Al-Bashir Governmental Hospital and Jamil Al-Totenji Governmental Hospital maternity clinics in Jordan. Binary logistic regression identified UTI predictors among pregnant women. Out of 536 participants, 34.3% of pregnant women were found to have UTIs. Amoxicillin/clavulanate demonstrated the highest level of resistance among the tested antibiotics and 42.4% of the pregnant participants adhered to the prescribed antibiotic regimen. Several factors were identified as contributing to an elevated risk of UTIs, including elevated body mass index (BMI) (*P* = .011), utilization of dry toilet paper (*P* = .023), frequent utilization of public restroom facilities (*P* < .001), delayed urination (*P* < .001), nonuse of cotton underwear (*P* = .019), frequent sexual activity (*P* < .001), lack of postcoital urination (*P* < .001), and inadequate genital hygiene before (*P* < .001) and after (*P* < .001) intercourse (including the husband’s). However, the use of wet wipes was the only factor associated with recurrent UTIs (*P* = .037). Emphasizing hygiene practices and managing BMI could play pivotal roles in reducing UTI risks among pregnant women.

## 1. Introduction

Urinary tract infections (UTIs) are highly prevalent bacterial infections among women, predominantly occurring between the ages of 16 and 35.^[1]^ It is estimated that annually 10% of women experience UTIs, and a substantial percentage (40%–60%) will encounter at least 1 infection in their lifetime.^[2]^

Pregnant women are particularly susceptible to UTIs, making it the most common type of infection during pregnancy. Around 10% of pregnant women are affected by UTIs, and all UTIs, including asymptomatic infections, should receive appropriate treatment.^[3]^ Asymptomatic bacteriuria is characterized by the presence of bacteria in the urine without exhibiting symptoms, occurs in 2% to 7% of pregnant women, usually occur early in their pregnancy. Around of 35% of symptomatic bacteriuria cases if left untreated may develop to a symptomatic UTI and/or pyelonephritis during pregnancy, which can be challenging and often necessitates hospitalization.^[4]^ Although the prevalence of asymptomatic UTIs in pregnant women varies, UTIs contribute to 20% of antenatal morbidity in Jordan.^[5]^ It is important to note that asymptomatic bacteriuria generally does not require treatment, except in pregnant women.^[1]^

Managing UTIs during pregnancy is of utmost importance owing to the possible adverse outcomes that can arise for both the expectant mother and the developing fetus. The presence of untreated or recurrent UTIs in expectant mothers can lead to various complications, including but not limited to pyelonephritis, preterm labor, low birth weight, fetal death, chronic renal failure, pregnancy hypertension, anemia, and postpartum sepsis.^[6]^ Furthermore, UTIs that occur during pregnancy have been linked to unfavorable outcomes for the developing fetus, such as intrauterine growth restriction and an elevated likelihood of neonatal morbidity and mortality.^[7]^

Previous literature reported several hygiene-related factors associated with UTI occurrence in pregnant women. For instance, increased intercourse frequency, infrequent underwear changes, poor hand hygiene and inadequate genital drying after voiding.^[8]^ Other practices like wearing tight clothes, using bath salts, fragrant soaps, and scented tissues have limited research to support direct comparisons.^[5]^

The urinary tract undergoes physical, hormonal, and functional alterations during pregnancy, which result in urine stasis and an elevated risk of microbial ascent from the bladder to the ureters.^[9]^ This is due to the fact that pregnant women are more susceptible to UTIs due to immunological changes, a naturally short urethra, and other factors.^[9]^ Previous studies in Jordan demonstrated limited knowledge and practices concerning medications utilization and antibiotic resistance among different populations.^[10–12]^ Despite several studies conducted on UTIs in Jordan regarding the prevalence and antimicrobial pattern, there remains a research gap in several important aspects. Existing studies have primarily focused on specific populations such as children, particular residential areas, or the general population.^[13–15]^ While a recent study by Matalka et al (2021) examined uropathogens and antimicrobial susceptibility in pregnant women with UTIs, there is still limited data on the prevalence of symptomatic and asymptomatic UTIs, risky practices contributing to UTIs among pregnant women, and recurrence rates.^[3]^ Therefore, this current study aimed to address these gaps by providing valuable insights into the prevalence rate of UTIs among pregnant women in Jordan, identifying potential risk practices that contribute to UTIs, examining the antimicrobial susceptibility, evaluating the adherence rate to antibiotic treatment regimens for managing UTIs, and assessing the recurrence rates of UTIs among pregnant women.

## 2. Methods

### 2.1. Study design and participant recruitment

A prospective cohort study was conducted from January to July 2023 on pregnant women in Jordan. The cohort study design was preferred in order to examine recurrent UTIs among pregnant women.

Participants were recruited during routine visits to Al-Bashir Governmental Hospital and Jamil Al-Totenji Governmental Hospital maternity clinics in Jordan. Al-Bashir Hospital is considered to be the main governmental hospital in Amman, the capital of Jordan. Established on August 22, 2001, the hospital serves a population of approximately 100,000 people. Dr Jamil Al-Tutanji Hospital is the main governmental hospital in Sahab area of Amman, Jordan. Besides, these hospitals provide maternity and antenatal care services for pregnant women from different socioeconomic backgrounds. Consent forms were signed by willing participants. A concise overview of UTIs, symptoms, potential effects on the mother and fetus, and the importance of treatment adherence was provided.

### 2.2. Sample size

Based on recent research in Jordan, the estimated prevalence rate of UTIs among pregnant women is 21.5%.^[5]^ The formula used to estimate the minimum required sample size is mentioned below:


$$\begin{array}{l} n=Z^{2}P\left(1-P\right)/d^{2} \end{array}$$

n is the required sample size, *Z* is the *Z*-score, *P* is the estimate prevalence rate of UTI among pregnant women, and *d* is the margin of error. Using a confidence interval of 95% and margin of error of 0.05 the minimum required sample size was 260 pregnant women. However, we targeted larger sample size in order to enhance the statistical power of the study estimates.

### 2.3. Inclusion and exclusion criteria

Pregnant women at any gestational age were eligible for inclusion in the research, as were those who willingly provided informed consent to contribute to the study’s objectives. On the other hand, individuals with a history of renal diseases or anatomical abnormalities in the urinary system were excluded from participation. Additionally, to ensure robust and accurate data collection, individuals with incomplete data or missing information in the administered questionnaires were also excluded from the study.

### 2.4. Study procedure

A convenience sampling technique was used for the recruitment of the participating women. Pregnant women visiting the maternity clinics and meeting the inclusion criteria were invited to participate in the study, and those who voluntarily accepted to take part were included after signing the informed consent.

The study procedure encompassed various stages of data collection and analysis. A structured questionnaire was employed to gather comprehensive information, including demographics, gynecologic and obstetric data, medical history, UTI signs, and risky practices. The questionnaire utilized close-ended questions and multiple-choice questions format. In addition, participants provided urine samples that were cultured to detect the presence of bacteria. The definition of UTI was established as the presence of a minimum of 100,000 organisms/mL of urine in asymptomatic patients, or >100 organisms/mL with pyuria in symptomatic patients. For those diagnosed with UTIs, pregnant women were prescribed antibiotics based on sensitivity results, adhering to treatment duration aligned with clinical guidelines, with a strong emphasis on treatment adherence. Subsequent to treatment, adherence was monitored through phone calls and a medication adherence report scale (MARS) self-report questionnaire, whereby a score of ≥ 80% on the aggregated MARS-5 denoted satisfactory adherence. A follow-up urine sample was collected posttreatment to evaluate efficacy and clearance of UTIs.

### 2.5. Questionnaire piloting

The questionnaire tool was evaluated by 3 expert pharmacists before starting the study. Moreover, before conducting the full study, the questionnaire tool was piloted on a small number of the targeted study population to ensure its clarity and understandability. The study participants confirmed that questionnaire items are clear and easy to complete.

### 2.6. Ethics approval

Ethical approval was obtained from the Ethics Committees of the Jordanian Ministry of Health (Reference no.: IRB/4033).

### 2.7. Data analysis

Data were analyzed using SPSS Version 28 (IBM Corp, Armonk). The normality of continuous variables was examined using histogram, skewness, and kurtosis measures. Normally distributed variables were presented as mean and standard deviation, while the median and interquartile range (IQR) were used to present non-normally distributed continuous variables. Differences in UTI-associated categorical variables were examined using Chi-squared test. Binary logistic regression identified UTI predictors among pregnant women. Besides, in order to control for potential confounding variables (age and BMI), multinomial logistic regression analysis was performed. The MARS questionnaire consisted of 6 inquiries designed to assess behaviors linked to nonadherence, each evaluated using a 5-point Likert scale. The accumulated scores were then computed, yielding a composite score ranging from 6 to 30, wherein higher scores denoted stronger adherence tendencies. For this study, a cutoff of 80% was applied, categorizing patients achieved total scores of ≥24 as adherent. A confidence interval of 95% (*P* < .05) was applied to represent the statistical significance of the results, and the level of significance was predetermined as 5%.

## 3. Results

### 3.1. Patients’ baseline characteristics

A total of 536 pregnant women participated in this study. Participants baseline characteristics and obstetric data are presented in Table 1. The ages of the participants exhibited an average age of 28 years, while the standard deviation was 5.3. It was observed that a significant proportion of women (54.30%) exhibited a body mass index (BMI) within the normal range. However, many participants exhibited higher body weight, with 19.9% classified as obese, and an additional 22.8% classified as overweight. The study revealed that 3 quarters of the participants 73.7%, were unemployed and 58.6%, had attained a high school education as their highest level of academic achievement. A total of 24.6% of the participants were identified as current smokers, whereas an additional 23% reported having ceased smoking. The findings indicate that a significant proportion (80%) of the research sample had monthly incomes of 500 JOD or lower.

**Table 1:** Patients’ demographic characteristics.

Variable	Frequency	Percentage	
Mean age (standard deviation) years	28.0 (5.3) yr		
BMI category			
Underweight	16	3.0	
Normal	291	54.3	
Overweight	122	22.8	
Obese	107	19.9	
Employment status			
Employed	141	26.3	
Unemployed	395	73.7	
Education			
Secondary school or lower	314	58.6	
Diploma	133	24.8	
Bachelor degree	86	16.0	
Postgraduate	3	0.6	
Smoking status			
None-smoker	280	52.2	
Current smoker	132	24.6	
Ex-smoker	124	23.1	
Monthly income			
500 JD and lower	429	80.0	
More than 500 JD	107	20.0	
Are you allergic to certain medicines (yes)	50	9.3	
Median number of pregnancies (whether completed or not)	4.0 (3.0–5.0)		
Method of delivery in the last pregnancy			
Normal delivery	358		66.9
Caesarean section	177		33.1
Median number of births after 24 wk	3.0 (2.0–4.0)		
Current mean gestational age	26.4 (5.1) yr		
Abortion history (yes)	259		48.3
Method of contraception			
Birth control pills	184		34.3
IUD	141		26.3
Condom	110		20.5
Insulation	73		13.6
Implant	28		5.2

BMI = body mass index, IUD = intrauterine device.

The data set exhibited an interquartile range spanning from 3.0 to 5.0 pregnancies, with a median value of 4.0. The median number of deliveries that occurred after 24 weeks fell within the range of 2.0 to 4.0. The mean gestational age of the respondents at the time of the study was 26.4 weeks, with a standard deviation of 5.1 weeks. The majority of women (66.9%) underwent a vaginal delivery for their most recent pregnancy, whereas approximately 1/3rd (33.1%) opted for a cesarean section.

A total of 48.3% of the participants indicated that they had undergone an abortion in the past. The utilization rates of contraceptive methods among women were as follows: 34.3% relied on pills, 26.3% used intrauterine devices (IUDs), 20.5% opted for condoms, 13.6% employed barrier methods, and 5.2% utilized implants.

### 3.2. General practices during pregnancy

The data presented in Table 2 provides insights into the daily behavioral patterns exhibited by pregnant women. According to the findings, a majority of the participants, specifically 60.1%, reported the utilization of cotton underwear. A smaller proportion of women reported wearing nylon (17.5%) and polyester (12.1%) underwear. A significant majority of participants (87.1%) reported a practice of changing their undergarments more frequently than 3 times per week. However, a mere 12.9% of women reported 1 to 3 times changing their underwear on a weekly basis.

**Table 2:** General practices during pregnancy.

Variable	Frequency	Percentage
The type of underwear used most of the time		
Cotton	322	60.1
Nylon	94	17.5
Polyester	65	12.1
Any type but tight fitting	46	8.6
Other	9	1.7
The number of times you change your underwear		
1 to 3 times a week	69	12.9
More than 3 times a week	467	87.1
How do you clean the sensitive area after urinating		
Rinse tube	308	57.5
Fixed bidet	179	33.4
Wet wipes	14	2.6
Dry toilet paper	27	5.0
Other	8	1.5
The direction of washing sensitive areas after urination		
From front to back	505	94.2
From back to front	31	5.8
Delayed voluntary or intentional voiding of urine		
I usually delay passing urine	214	39.9
I don’t delay passing urine at all	322	60.1
Use of public restrooms or work restrooms		
Frequently	58	10.8
When urgently needed	320	59.7
I never use it	158	29.5
Using deodorants or vaginal perfumes		
I use it	232	43.3
I don’t use it	304	56.7
Average number of sexual intercourses during pregnancy		
1 to 2 times a week	323	60.4
More than twice a wk	212	39.6
Washing the wife’s intimate area before sexual intercourse		
Yes	356	66.4
No	26	4.9
Sometimes	154	28.7
The husband washed the intimate area before intercourse		
Yes	262	48.9
No	39	7.3
Sometimes	235	43.8
Washing the wife’s intimate area after sexual intercourse		
Within 15 min	333	62.1
After 15 min or more	203	37.9
Emptying urine after sexual intercourse		
Within 15 min	389	72.6
After 15 min or more	147	27.4
Number of visits to the gynecologist and obstetrician		
More than once a month	43	8.0
Once a month	438	81.7
Once every 2 or 3 mo or more	55	10.3

A significant proportion of individuals (57.5%) indicated their utilization of a rinse tube, a customary method observed in numerous societies for the purpose of cleansing following urination. A mere 33.4% of the participants opted to utilize the installed bidets, whereas the remaining 2.6% and 5.0% of individuals resorted to employing wet wipes and dry toilet paper, respectively.

The majority of individuals (94.2%) reported adhering to the recommended practice of washing from front to back, a technique designed to minimize the risk of bacterial transmission from the rectal cavity to the urethra. Merely a limited fraction, specifically 5.8% of participants, indicated the practice of laundering their garments in an inverted manner.

A mere 29.5% of participants indicated that they abstain from utilizing public restroom facilities. Additionally, it was found that 43.3% of the participants reported using deodorant or vaginal perfume during their pregnancy.

More than half of the participants (60.4%) reported engaging in sexual activity on a weekly basis, specifically between once and twice per week. Conversely, a minority (39.6%) indicated a higher frequency of sexual activity beyond this range. The study also examined both pre- and postcoital hygiene practices. According to the findings, a majority of women (66.4%) and half of the partners (48.9%) reported cleansing genitalia within a time frame of 15 minutes prior to sexual activity. Moreover, a significant proportion of individuals, specifically 62.1%, indicated that they engaged in urination within a time frame of 15 minutes following sexual activity.

The overwhelming majority of participants (81.7%) reported a monthly frequency of visits to their gynecologist or obstetrician. A smaller proportion (8.0%) indicated more frequent visits, while a 10.3% reported less frequent visits, occurring once every 2 or 3 months.

### 3.3. Urinary tract infection during pregnancy

A significant portion of pregnant women within the research cohort experience UTIs, as evidenced by the positive urine culture results for UTIs, which accounted for 34.3% (n = 184) of the total. Table 3 presents essential statistical data regarding the associated symptoms reported by affected individuals. A comprehensive examination was conducted on various symptoms associated with UTIs during pregnancy. UTI infection symptoms were more common across pregnant women with UTI compared with pregnant women without UTI (*P* < .001). The most prevalent symptom reported by individuals was an abrupt need to urinate, with a frequency of 60.1%. This was closely followed by symptoms of vomiting (50.9%), nausea (46.5%), and vaginal itching (44.2%). During sexual activity, a significant proportion of individuals, specifically 43.6%, reported experiencing discomfort in the lower abdomen or vagina. Additionally, 39.0% of participants reported experiencing pain in the loin region. The aforementioned signs and symptoms are commonly observed in individuals who are not pregnant and are indicative of a UTI. Nevertheless, the identification of a UTI during pregnancy can pose challenges as its symptoms frequently overlap with those of other pregnancy-related conditions. Hence, it is imperative to possess knowledge of these indicators in order to facilitate timely identification and intervention.

**Table 3:** Urinary tract infection symptoms during pregnancy.

Symptoms	Overall		Pregnant with UTI	Pregnant without UTI	*P*-value
	Frequency	Percentage	Percentage	Percentage	
Urgent need to urinate	322	60.1	77.4	50.9	<.001
Vomiting	273	50.9	61.8	45.1	<.001
Nausea	249	46.5	57.5	40.6	<.001
Vaginal itching	237	44.2	61.8	34.9	<.001
Pain in the lower abdomen and in the vagina during intercourse	233	43.6	58.9	35.5	<.001
Pain in the loin	209	39.0	60.8	27.4	<.001
Thick vaginal secretions	205	38.3	58.9	27.4	<.001
Unknown heat source	198	36.9	54.3	27.7	<.001
Burning during urination	190	35.4	59.7	22.6	<.001
Urinating more than 8 times a day	179	33.4	54.8	22.0	<.001
Difficulty or pain during urination	165	30.8	61.3	14.6	<.001
Urinary retention	114	21.3	38.2	12.3	<.001
Presence of blood with urine	52	9.7	16.1	6.3	<.001

A significant proportion of women, specifically 38.3%, reported the occurrence of thick vaginal discharges, while 36.9% reported experiencing internal sensations of overheating. A prevalent manifestation of UTIs is a sensation of burning during urination, as indicated by a reported incidence of 35.4% among individuals. Additional prevalent symptoms encompassed frequent urination exceeding 8 occurrences per day (33.4%) and the presence of discomfort or pain during urination (30.8%). Urinary retention, characterized by the inability to fully void the bladder, was observed in 21.3% of the individuals comprising the sample population. A total of 9.7% of individuals indicated the presence of hematuria, or blood in their urine.

**Figure 1.**
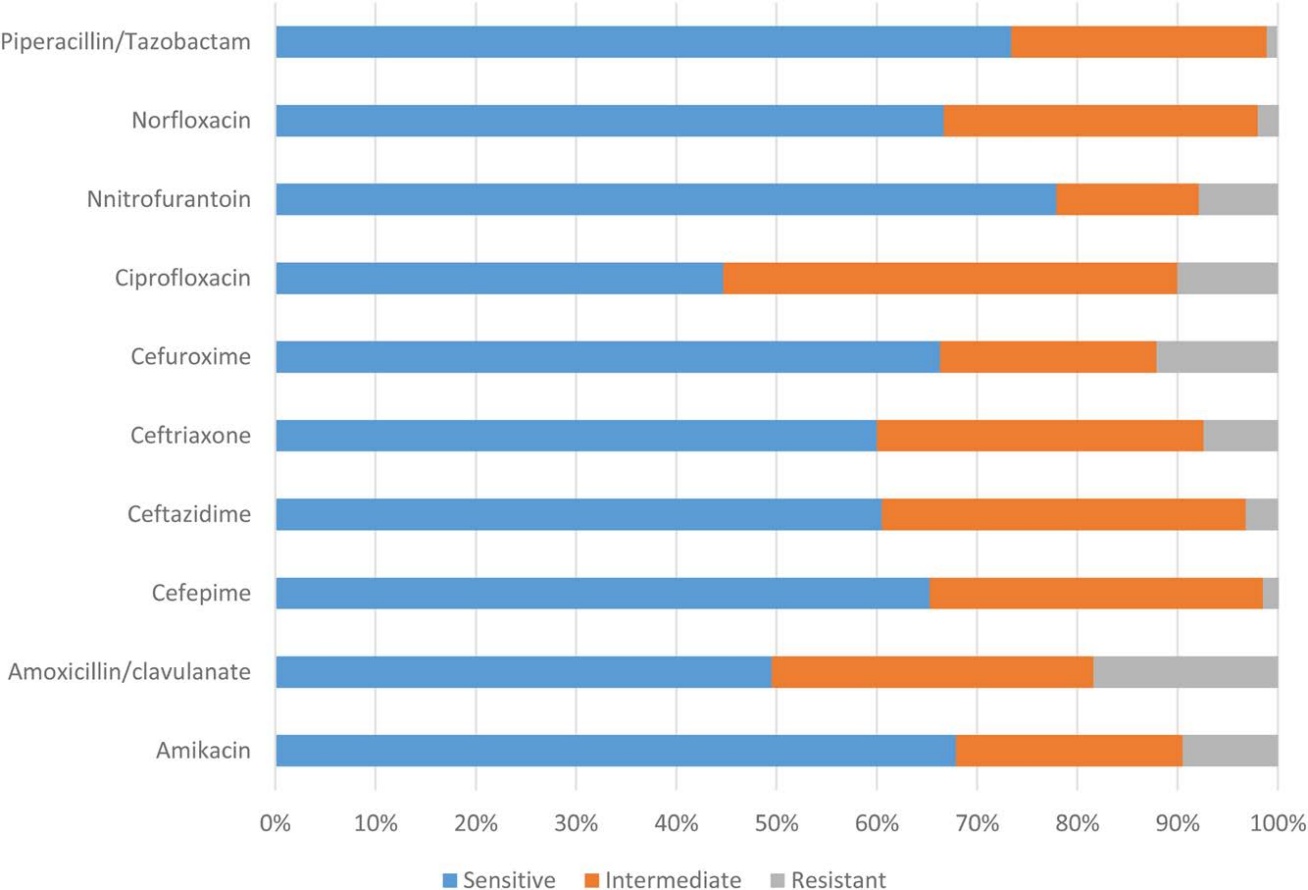
Antibiotic resistance profile.

The observed sensitivity rates of Nitrofurantoin (77.9%) and Piperacillin/Tazobactam (73.4%) indicate that these antimicrobial agents could be viable treatment options for UTIs occurring during pregnancy, specifically those caused by the identified bacterial strains (Fig. 1).

Amikacin demonstrates high sensitivity rate of 67.9%, also noted for Cefuroxime, Cefepime, Ceftazidime, and Ceftriaxone making them notable antibiotic options for the treatment of UTIs caused by the isolated bacteria. Nevertheless, the sensitivity rate exhibited by Amoxicillin/clavulanate, which is below 50%, suggest that its efficacy in treating UTIs caused by these organisms may be limited. Approximately 50% of the bacteria that were isolated exhibited either intermediate resistance or complete resistance to the antibiotic ciprofloxacin.

### 3.4. Medications adherence profile

The average medication adherence rate was calculated to be 19.3 out of a maximum score of 25. Using a cutoff point of 80% (the patients had total scores ≥ 20 were considered adherent), the estimated adherence rate in our study was about 42.4% (n = 78). The scores of the participants suggest that they are adhering to the instructions provided by their physicians regarding the consumption of prescribed medications.

### 3.5. Predictors of UTI infections among pregnant women

A binary logistic regression model was employed to examine the probability of acquiring a UTI or experiencing recurrent UTIs. Table 4 displays the odds ratios and p-values associated with each predictor variable.

**Table 4:** Factors contributing to antimicrobial resistance among pregnant women with UTI.

Variable	Odds ratio of UTI infection (95% confidence interval)	*P*-value	Adjusted odds ratio of UTI infection (95% confidence interval)†	*P*-value	Odds ratio of UTI recurrence (95% confidence interval)	*P*-value	Adjusted odds ratio of UTI recurrence (95% confidence interval)†	*P*-value	
Age category									
<28 yr (reference category)	1.00				1.00				
28 yr and above	0.96 (0.67–1.37)	.833			2.12 (0.62–7.32)	.233			
BMI category									
Underweight (reference category)	1.00				1.00				
Normal	0.72 (0.50–1.03)	.069			0.47 (0.14–1.62)	.233			
Overweight	0.813 (0.53–1.25)	0.349			3.13 (0.93–10.53)	.065			
Obese	1.75 (1.14–2.70)	.011*			0.99 (0.26–3.84)	.991			
Employment status									
Employed (reference category)	1.00		1.00		1.00		1.00		
Unemployed	1.24 (0.82–1.87)	.310	1.21 (0.80–1.84)	.366	1.52 (0.32–7.25)	.597	1.65 (0.34–7.93)	.531	
Education									
Secondary school or lower (reference category)	1.00		1.00		1.00		1.00		
Diploma	0.79 (0.52–1.21)	.280	0.80 (0.52–1.24)	.328	2.90 (0.87–9.72)	.084	3.43 (0.90–13.05)	.071	
Bachelor degree	1.01 (0.62–1.64)	.969	0.92 (0.55–1.53)	.739	1.09 (0.23–5.24)	.919	1.33 (0.24–7.37)	.748	
Postgraduate	–				–				
Smoking status									
None-smoker (reference category)	1.00		1.00		1.00		1.00		
Current smoker	1.43 (0.95–2.14)	.085	1.57 (1.02–2.42)	.042*	0.80 (0.21–3.09)	.749	0.65 (0.16–2.69)	.556	
Ex-smoker	1.15 (0.76–1.74)	.523	1.37 (0.88–2.14)	.170	0.54 (0.11–2.57)	.441	0.51 (0.10–2.62)	.422	
Monthly income									
500 JOD and lower (Reference category)	1.00		1.00		1.00		1.00		
More than 500 JOD	0.93 (0.60–1.44)	.74	–		0.85 (0.19–3.90)	.837	–		
Are you allergic to certain medicines									
No (reference category)	1.00		1.00		1.00		1.00		
Yes	1.17 (0.70–1.97)	.543	–		1.31 (0.43–3.93)	.634	–		
Method of delivery in the last pregnancy									
Normal delivery (reference category)	1.00		1.00		1.00		1.00		
Caesarean section	1.21 (0.85–1.71)	.294	1.52 (1.03–2.25)	.035*	1.77 (0.59–5.31)	.307	1.79 (0.53–6.08)	.350	
The type of underwear used most of the time									
Cotton	0.65 (0.45–0.93)	.019*	0.23 (0.06–0.94)	.040*	1.20 (0.37–3.93)	.766	0.25 (0.02–2.75)	.257	
Nylon	1.28 (0.81–2.02)	.297	0.35 (0.08–1.50)	.157	1.35 (0.35–5.28)	.663	0.35 (0.03–4.40)	.414	
Polyester	1.39 (0.82–2.37)	.218	0.38 (0.09–1.67)	.199	–		–		
Any type but tight fitting	1.11 (0.60–2.08)	.737	0.28 (0.06–1.29)	.103	0.86 (0.10–7.08)	.887	0.19 (0.01–4.14)	.294	
The number of times you change your underwear									
1 to 3 times a week	1.00		1.00		1.00		1.00		
More than 3 times a week	0.84 (0.50–1.42)	.520	–		–		–		
How do you clean the sensitive area after urinating									
Rinse tube	0.94 (0.66–1.34)	.730	1.43 (0.28–7.22)	.667	1.75 (0.51–6.05)	.374	0.14 (0.01–2.58)	.185	
Fixed bidet	0.86 (0.59–1.26)	.429	1.29 (0.25–6.60)	.764	0.18 (0.02–1.39)	.100	0.03 (0.00–0.91)	.044*	
Wet wipes	1.43 (0.49–4.17)	.518	2.09 (0.31–14.31)	.452	6.48 (1.12–37.66)	.037*	0.77 (0.03–22.00)	.877	
Dry toilet paper	2.47 (1.13–5.40)	.023*	3.23 (0.55–19.17)	.197	–		–		
The direction of washing sensitive areas after urination									
From front to back	1.00		1.00		1.00		1.00		
From back to front	1.83 (0.88–3.79)	.103	1.85 (0.89–3.86)	.101	2.37 (0.47–11.98)	.297	2.57 (0.49–13.39)	.263	
Delayed voluntary or intentional voiding of urine									
I usually delay passing urine	1.00		1.00		1.00		1.00		
I don’t delay passing urine at all	0.47 (0.33–0.68)	<.001***	–		1.56 (0.48–5.11)	.463	1.38 (0.41–4.63)	.600	
Use of public restrooms or work restrooms									
Frequently	1.00		1.00		1.00		1.00		
When urgently needed	1.96 (1.34–2.85)	<.001***	0.66 (0.38–1.17)	.153	0.78 (0.23–2.73)	.701	1.81 (0.21–15.30)	.587	
I never use it	0.28 (0.18–0.45)	<.001***	0.21 (0.11–0.40)	<.001***	2.45 (0.61–9.81)	.206	4.40 (0.41–47.15)	.221	
Using deodorants or vaginal perfumes									
I use it	1.00		1.00		1.00		1.00		
I don’t use it	0.75 (0.52–1.07)	.108	–		0.44 (0.13–1.51)	.192	0.44 (0.13–1.53)	.197	
Average number of sexual intercourse during pregnancy									
1 to 2 times a week	1.00		1.00		1.00		1.00		
More than twice a week	2.05 (1.43–2.94)	<.001***	–		0.60 (0.18–1.98)	.404	0.641 (0.19–2.13)	.467	
Washing the wife’s intimate area before sexual intercourse									
Yes	1.00		1.00		1.00		1.00		
No	1.95 (0.88–4.29)	.098	2.93 (1.31–6.59)	.009	–		–		
Sometimes	2.82 (1.91–4.15)	<.001***	3.09 (2.08–4.61)	<.001***	0.66 (0.19–2.28)	.510	0.67 (0.19–2.38)		.536
The husband washed the intimate area before intercourse									
Yes	1.00		1.00		1.00		1.00		
No	2.35 (1.22–4.53)	.011*	4.58 (2.27–9.23)	<.001***	–		–		
Sometimes	2.58 (1.79–3.72)	<.001***	3.32 (2.23–4.93)	<.001***	1.55 (0.45–5.36)	.486	1.42 (0.39–5.10)	.595	
Washing the wife’s intimate area after sexual intercourse									
Within 15 min	1.00		1.00		1.00		1.00		
After 15 min or more	3.74 (2.59–5.40)	<.001***	1.22 (0.12–12.15)	.867	0.72 (0.22–2.33)	.586	0.79 (0.24–2.61)	.696	
Emptying urine after sexual intercourse									
Within 15 min	1.00		1.00		1.00		1.00		
After 15 min or more	2.53 (1.73–3.72)	<.001***	2.61 (0.16–42.03)	.500	0.54 (0.14–2.06)	.364	0.59 (0.15–2.28)	.440	
Number of visits to the gynecologist and obstetrician									
More than once a month	1.00		1.00		1.00		1.00		
Once a month	1.50 (0.92–2.43)	.101	1.43 (0.72–2.82)	.307	0.84 (0.17–4.07)	.828	–		
Once every 2 or 3 mo or more	0.63 (0.33–1.19)	.156	0.94 (0.38–2.31)	.895	2.37 (0.47–11.98)	.297	–		

BMI = body mass index.

† Adjusted for age and BMI.

* *P* < .05.

*** *P* < .001.

Individuals with an elevated BMI exhibit a heightened propensity for experiencing UTI during the gestational period in comparison to those with a lower BMI (OR = 1.75, CI = 1.14–2.70, *P* = .011). Nevertheless, the variable of BMI did not exhibit a statistically significant association with the recurrence of UTI. A significant association was observed between the utilization of dry toilet paper for cleansing the perineal region following urination and the incidence of UTIs (OR = 2.47, CI = 1.13–5.40, *P* = .023). Nevertheless, there is an association between the recurrence of UTIs and the utilization of moist wipes for personal hygiene (OR = 6.48, CI = 1.12–37.66, *P* = .037). Furthermore, the findings showed that pregnant women who opt for cotton underwear are less likely to experience UTIs (OR = 0.65, CI = 0.45–0.93, *P* = .019). Moreover, multinomial logistic regression analysis confirmed that current smokers, those whom las delivery method was cesarean section, those whom sometimes clean their intimate area before sexual intercourse, and those whom their husbands do not wash the intimate area before intercourse are more likely to experience UTIs (*P* < .05). Besides, those who clean the sensitive area after urinating using fixed bidet were less likely to have recurrent UTIs compared to others (*P* < .05).

Women who frequently utilized public or workplace restrooms exhibited a significantly elevated susceptibility to UTIs in comparison to those who abstained from such usage. Nevertheless, this variable did not exhibit a statistically significant correlation with the occurrence of recurrent UTIs. The frequency of sexual activity exceeding twice weekly significantly elevates the likelihood of UTI development in pregnant women. Nevertheless, a lack of statistically significant correlation was observed between the frequency of sexual encounters and the reoccurrence of UTIs in pregnant women. The likelihood of contracting a UTI was found to be significantly increased when either the female or her male partner failed to consistently cleanse the genital area prior to engaging in sexual intercourse. Nevertheless, the analysis did not reveal any statistically significant correlation between repeat episodes of UTI. UTIs exhibited a higher prevalence among women who habitually engaged in the practice of delaying urination, in contrast to those who refrained from postponing urination altogether. Nevertheless, this variable did not exhibit a statistically significant correlation with the occurrence of recurrent UTIs.

## 4. Discussion

The findings of this study revealed a notably high prevalence of UTIs (34.3%) among pregnant women, which highlights the significance of this health concern during pregnancy. The prevalence rate is comparable to Nepal (37.8%),^[16]^ with higher prevalence rates reported in Libya (49.3%) and Saudi Arabia (53.5%).^[17,18]^ Conversely, lower rates were found in Iran (13.1%), India (13.4%), and Egypt (29%).^[19–21]^ These variations stem from differences in study parameters, sample sizes, social habits, hygiene standards, and education levels across different regions and countries.^[22]^

*Escherichia coli* is the most prevalent causative organism in UTIs, as reported by previous research findings.^[3,23,24]^ It is noteworthy that amoxicillin/clavulanate in the present study exhibited the highest resistance among the tested antibiotics, underscoring the importance of judicious use of antibiotics to combat the issue of antibiotic resistance in UTI management, particularly during pregnancy. In Jordan, antibiotics are readily accessible to the public, as they are dispensed without a prescription from most pharmacies.^[25]^ A previous study conducted in Jordan reported that over 70% of pharmacies provide antibiotics without requiring a prescription. Pharmacists themselves stated that they would dispense antibiotics without a prescription for conditions like upper respiratory tract infections, diarrhea, and UTI.^[26]^ Notably, amoxicillin/clavulanate emerged as the most frequently self-medicated antibiotic among the public.^[27,28]^ This unrestricted access to antibiotics and the practice of self-medication could contribute to antibiotic resistance and other adverse health consequences, emphasizing the need for better regulation and awareness campaigns to promote responsible antibiotic use in the community.^[29]^

The study’s findings indicate that less than half of pregnant women, specifically 42.4%, adhered to the prescribed antibiotic regimen. The importance of completing a full course of antibiotics for effective treatment and prevention of antibiotic resistance has been emphasized in several studies.^[12,30,31]^ Therefore, the importance of engaging in a comprehensive dialogue between pregnant women and their healthcare professionals regarding the necessity of adhering to prescribed antibiotic regimens and the potential consequences of noncompliance cannot be overstated.

The regression analysis findings indicate that pregnant women with an elevated BMI face an increased susceptibility to UTIs during pregnancy; women in the obese BMI category had an odds ratio of 1.75 (*P* = .011), indicating an increased likelihood of having UTI in comparison with normal weight women. Similarly, Rejali et al (2015) found a significant relationship between urinary infection and BMI,^[32]^ while Balachandran et al (2022) in contrast found that there was no significant association between BMI and UTI.^[33]^

The study additionally discovered robust associations between specific forms of personal hygiene and the incidence of UTIs. The utilization of wet wipes for personal hygiene has been found to be significantly associated with an increased likelihood of recurring UTIs, as indicated by an odds ratio of 6.48 and a *P*-value of .037. As reported by Crann et al (2018), participants who reported using wet wipes had nearly 60% higher odds of experiencing a UTI (OR = 1.6, 95% CI: 1.1–2.3, *P* = .02).^[34]^ On the other hand, the utilization of dry toilet paper has been found to exhibit a positive correlation with the likelihood of experiencing a UTI in individuals who have not previously encountered such an infection. This contrasts with the findings of Jelly et al’s study in 2022, where over 50% of participants who didn’t dry the perineal area with toilet paper after washing were linked to increased UTI occurrences.^[35]^

Furthermore, the research conducted revealed a positive correlation between frequent utilization of public or shared restroom facilities and an increased susceptibility to UTIs. The utilization of public bathrooms has been found to be significantly correlated with a 1.96-fold higher likelihood of contracting a disease (*P* < .001). Public toilets, lacking proper regulation and standards, become breeding grounds for germ transmission, spreading urogenital infections despite seemingly clean appearances.^[36]^ This conclusion is also supported by Magistro et al (2015), Johnson et al (2005), and Cooke et al (2010).^[36–39]^ On the other hand, Women refraining from timely voiding has been identified as a significant UTI risk in this study. The act of delaying urination, a behavior known as delayed voiding, has been highlighted by Jagtap et al (2022) as a potential risk factor for UTIs.^[40]^ Additionally, it was noticed that cotton has protective effect from UTI. Cotton-based innerwear absorbs perspiration better than synthetic options, reducing the risk of UTIs.^[36,41–43]^

Our study found that other genital hygiene practices, such as frequent coitus, not urinating after coitus, and inadequate washing of genitals before and after coitus (including the husband’s), were associated with an increased risk of UTIs, consistent with findings from other studies.^[5,8,44,45]^

This study has some limitations. Firstly, researcher relied on recorded urine culture results and antimicrobial susceptibility data, without direct involvement in the testing process. Secondly, reliance on self-reported data may introduce bias, potentially resulting in underreporting of risk factors, symptoms, or nonadherence rate.

## 5. Conclusion

The study’s findings revealed a notable prevalence of UTIs in pregnant women, with Amoxicillin/clavulanate displaying the highest resistance among tested antibiotics. Furthermore, the study indicated that pregnant women’s adherence to prescribed antibiotic regimens is somewhat limited. Promoting awareness among pregnant women regarding the importance of completing antibiotic courses is vital. Regression analysis further illuminated the significant roles played by BMI and hygiene practices in influencing UTI incidence. Thus, emphasizing proper hygiene practices, along with managing BMI, could potentially reduce the risk of UTIs.

## Acknowledgments

We would like to acknowledge Princess Nourah bint Abdulrahman University Researchers Supporting Project number (PNURSP2025R483), Princess Nourah bint Abdulrahman University, Riyadh, Saudi Arabia.

## Author contributions

**Conceptualization:** Asma Bakleezi, Esra’ O. Taybeh.

**Data curation:** Asma Bakleezi, Abdallah Y. Naser.

**Formal analysis:** Abdallah Y. Naser.

**Investigation:** Asma Bakleezi, Esra’ O. Taybeh, Abdalrahman Binodeh, Alaa A. Alsharif, Meshari Alhamed, Abdallah Y. Naser.

**Methodology:** Esra’ O. Taybeh, Abdallah Y. Naser.

**Project administration:** Esra’ O. Taybeh.

**Resources:** Asma Bakleezi, Esra’ O. Taybeh, Abdalrahman Binodeh, Alaa A. Alsharif, Meshari Alhamed, Abdallah Y. Naser.

**Software:** Abdallah Y. Naser.

**Supervision:** Esra’ O. Taybeh, Abdallah Y. Naser.

**Validation:** Esra’ O. Taybeh, Abdallah Y. Naser.

**Visualization:** Asma Bakleezi, Esra’ O. Taybeh, Abdallah Y. Naser.

**Writing – original draft:** Asma Bakleezi, Esra’ O. Taybeh, Abdalrahman Binodeh, Alaa A. Alsharif, Meshari Alhamed, Abdallah Y. Naser.

**Writing – review & editing:** Asma Bakleezi, Esra’ O. Taybeh, Abdalrahman Binodeh, Alaa A. Alsharif, Meshari Alhamed, Abdallah Y. Naser.
